# Supplementary material for: Estimating access to surgical care: A community centered national household survey from Pakistan
Source: PLOS Glob Public Health. 2023 Nov 15;3(11):e0002130. doi: 10.1371/journal.pgph.0002130 (PMC10651040; doi:10.1371/journal.pgph.0002130)
Supplement: S1 Text — (PDF) [file pgph.0002130.s001.pdf]

# Annex 1: Household Survey Questionnaire

**Part 1: Registration**

Record ID

___________________________

Data entered by:

___________________________8uu

1. Date of interview
   ____________________
2. Province:
   - o Sindh
   - o Punjab
   - o KP
   - o Balochistan
   - o Islamabad
3. Latitude of house
   ____________________
4. Longitude of house
   ____________________
5. Nearby landmark to the house
   ____________________
6. Do you consent to participate in the study?
   - o Yes
   - o No
7. If you do not consent, then please share your reason.
   ______________________________________________________________________________
   ________________________________________________________________

**Part 2: Household Member Listing**

Record ID

_______________________________

Data entered by:

_______________________________

1. How many members live in this household?

   _______________________________

2. Enlist the sex and age of the household members starting from the oldest to the youngest.

| # | Sex | Age | # | Sex | Age |
|---|---|---|---|---|---|
| 1 | | | 14 | | |
| 2 | | | 15 | | |
| 3 | | | 16 | | |
| 4 | | | 17 | | |
| 5 | | | 18 | | |
| 6 | | | 19 | | |
| 7 | | | 20 | | |
| 8 | | | 21 | | |
| 9 | | | 22 | | |
| 10 | | | 23 | | |
| 11 | | | 24 | | |
| 12 | | | 25 | | |
| 13 | | | 26 | | |

**Part 3: C -section**

Record ID:

____________________________

Data Entered by:

____________________________

***Part 3a: Access to preferred hospital***

1. If anyone in the family would need a Caesarean section/C-section (operation needed to deliver baby), which hospital would you prefer to go to? ____________________________
2. What is a famous landmark near the hospital. ____________________________
3. Out of the following 9 modes of transport, how many methods would you use? Ambulance, public transport (bus/rickshaw), Car, Motorcycle, Bicycle, Boat, Animal transport, walking, being carried by someone else)
   - One
   - Two
   - Three

If one, skip question 5 and 6

If two, skip question 6

4. What would your first mode of transport be in order to reach the hospital
   - Ambulance
   - Public transport
   - Rickshaw
   - Own car
   - Taxi/rented car
   - Neighbour's car
   - Motorcycle
   - Bicycle
   - Boat
   - Animal transport
   - Walking
   - Being carried by someone else

5. What would your second mode of transport be in order to reach the hospital
   - Ambulance
   - Public transport
   - Rickshaw
   - Own car
   - Taxi/rented car

- Neighbour’s car
- Motorcycle
- Bicycle
- Boat
- Animal transport
- Walking
- Being carried by someone else

6. What would your third mode of transport be in order to reach the hospital
    - Ambulance
    - Public transport
    - Rickshaw
    - Own car
    - Taxi/rented car
    - Neighbour’s car
    - Motorcycle
    - Bicycle
    - Boat
    - Animal transport
    - Walking
    - Being carried by someone else
7. How long do you have to wait for arrangement of transport?

    ______________________________
8. After arrangement of transport, how long will it take you to reach the above mentioned hospital?

    ______________________________
9. How much will the transport cost?
    - 0-100 PKR
    - 100-200 PKR
    - 200-300 PKR
    - 300-400 PKR
    - 400-500 PKR
    - 500-1000 PKR
    - >1000 PKR
10. Is this much amount of money always readily available to you?
    - Yes
    - No

***Part 3b: Past experience***

11. In the last three years, has any household member required a Caesarean section/C-section (operation needed to deliver baby)?
    - Yes
    - No

If no, then skip to next section.

12. How long ago did they have the Caesarean section/C-section (operation needed to deliver baby)?
    - Less than 1 year
    - Less than 2 years but more than 1 year
    - Less than 3 years but more than 2 years.
13. Which hospital did you go to for this Caesarean section/C-section (operation needed to deliver baby)?
    - Same as above hospital mentioned in preference
    - Different Hospital
14. If it was a different hospital, what is the name of the hospital you went to?
    ______________________________
15. What is a famous landmark near this hospital
    ______________________________
16. What was the reason to choose this hospital?
    - Excellent doctors, nurses, and staff
    - Affordable treatment
    - Free medicines available
    - Focused on quality improvement
    - Referred by a friend/relative who also got treated here
    - My previous experience of treatment here was good
    - Referred by someone
    - Other reason
    - Don't know
    - No answer
17. If other reason, please explain
    ______________________________
18. After reaching the hospital, how much time did you wait before you were seen by a doctor?
    ______________________________
19. Quality at hospital

| | Not satisfied at all | Satisfied | Very satisfied |
|---|---|---|---|
| Were you satisfied that the doctor listened to you attentively? | | | |
| Were you satisfied with the doctor's knowledge and experience? | | | |
| Were you satisfied that the doctor talked to you with respect? | | | |
| Were you satisfied with the amount of time doctor spent with you? | | | |
| Overall, how satisfied were you with your | | | |

| | | | |
|---|---|---|---|
| experience at this hospital? | | | |

20. What is the patient’s condition now?
    - Completely treated
    - Short term complications
    - Long term complications
    - Death of the patient

**Part 4: Operative Fracture Repair**

Record ID:

___________________________

Data Entered by:

___________________________

***Part 4a: Access to preferred hospital***

1. If anyone in the family would need a Operative fracture repair (Operation to fix broken bones), which hospital would you prefer to go to?
   ____________________________
2. What is a famous landmark near the hospital.
   ____________________________
3. Out of the following 9 modes of transport, how many methods would you use? Ambulance, public transport (bus/rickshaw), Car, Motorcycle, Bicycle, Boat, Animal transport, walking, being carried by someone else)
    - One
    - Two
    - Three

If one, skip question 5 and 6

If two, skip question 6

4. What would your first mode of transport be in order to reach the hospital
    - Ambulance
    - Public transport
    - Rickshaw
    - Own car
    - Taxi/rented car
    - Neighbour’s car
    - Motorcycle
    - Bicycle
    - Boat
    - Animal transport
    - Walking
    - Being carried by someone else

5. What would your second mode of transport be in order to reach the hospital
    - Ambulance
    - Public transport
    - Rickshaw
    - Own car
    - Taxi/rented car
    - Neighbour's car
    - Motorcycle
    - Bicycle
    - Boat
    - Animal transport
    - Walking
    - Being carried by someone else
6. What would your third mode of transport be in order to reach the hospital
    - Ambulance
    - Public transport
    - Rickshaw
    - Own car
    - Taxi/rented car
    - Neighbour's car
    - Motorcycle
    - Bicycle
    - Boat
    - Animal transport
    - Walking
    - Being carried by someone else
7. How long do you have to wait for arrangement of transport?
    ______________________________
8. After arrangement of transport, how long will it take you to reach the above mentioned hospital?
    ______________________________
9. How much will the transport cost?
    - 0-100 PKR
    - 100-200 PKR
    - 200-300 PKR
    - 300-400 PKR
    - 400-500 PKR
    - 500-1000 PKR
    - >1000 PKR
10. Is this much amount of money always readily available to you?
    - Yes
    - No

***Part 4b: Past experience***

11. In the last three years, has any household member required a Operative Fracture Repair?
    - Yes
    - No

If no, then skip to the next section.

12. How long ago did they have the Operative Fracture Repair?
    - Less than 1 year
    - Less than 2 years but more than 1 year
    - Less than 3 years but more than 2 years.
13. Which hospital did you go to for this Operative Fracture Repair?
    - Same as above hospital mentioned in preference
    - Different Hospital
14. If it was a different hospital, what is the name of the hospital you went to?
    ______________________________
15. What is a famous landmark near this hospital
    _____________________________
16. What was the reason to choose this hospital?
    - Excellent doctors, nurses, and staff
    - Affordable treatment
    - Free medicines available
    - Focused on quality improvement
    - Referred by a friend/relative who also got treated here
    - My previous experience of treatment here was good
    - Referred by someone
    - Other reason
    - Don't know
    - No answer
17. If other reason, please explain
    _____________________________
18. After reaching the hospital, how much time did you wait before you were seen by a doctor?
    _____________________________
19. Quality at hospital

| | Not satisfied at all | Satisfied | Very satisfied |
|---|---|---|---|
| Were you satisfied that the doctor listened to you attentively? | | | |
| Were you satisfied with the doctor's knowledge and experience? | | | |
| Were you satisfied that the doctor talked to you with respect? | | | |

| | | | |
|---|---|---|---|
| Were you satisfied with the amount of time doctor spent with you? | | | |
| Overall, how satisfied were you with your experience at this hospital? | | | |

20. What is the patient's condition now?
    - Completely treated
    - Short term complications
    - Long term complications
    - Death of the patient

**Part 5: Laparotomy**

Record ID:

_____________________________

Data Entered by:

_____________________________

***Part 5a: Access to preferred hospital***

1. If anyone in the family would need a Laparotomy (Operation for stomach, appendix, intestinal disease), which hospital would you prefer to go to?
   ______________________________
2. What is a famous landmark near the hospital.
   ______________________________
3. Out of the following 9 modes of transport, how many methods would you use? Ambulance, public transport (bus/rickshaw), Car, Motorcycle, Bicycle, Boat, Animal transport, walking, being carried by someone else)
    - One
    - Two
    - Three

If one, skip question 5 and 6

If two, skip question 6

4. What would your first mode of transport be in order to reach the hospital
    - Ambulance
    - Public transport
    - Rickshaw
    - Own car
    - Taxi/rented car
    - Neighbour's car
    - Motorcycle

- Bicycle
- Boat
- Animal transport
- Walking
- Being carried by someone else

5. What would your second mode of transport be in order to reach the hospital
    - Ambulance
    - Public transport
    - Rickshaw
    - Own car
    - Taxi/rented car
    - Neighbour’s car
    - Motorcycle
    - Bicycle
    - Boat
    - Animal transport
    - Walking
    - Being carried by someone else
6. What would your third mode of transport be in order to reach the hospital
    - Ambulance
    - Public transport
    - Rickshaw
    - Own car
    - Taxi/rented car
    - Neighbour’s car
    - Motorcycle
    - Bicycle
    - Boat
    - Animal transport
    - Walking
    - Being carried by someone else
7. How long do you have to wait for arrangement of transport?
    ______________________________
8. After arrangement of transport, how long will it take you to reach the above mentioned hospital?
    ______________________________
9. How much will the transport cost?
    - 0-100 PKR
    - 100-200 PKR
    - 200-300 PKR
    - 300-400 PKR
    - 400-500 PKR
    - 500-1000 PKR

- >1000 PKR

10. Is this much amount of money always readily available to you?
    - Yes
    - No

***Part 5b: Past experience***

11. In the last three years, has any household member required a Laparotomy (Operation for stomach, appendix, intestinal disease)?
    - Yes
    - No

If no, then skip to next section.

12. How long ago did they have the Laparotomy (Operation for stomach, appendix, intestinal disease)?
    - Less than 1 year
    - Less than 2 years but more than 1 year
    - Less than 3 years but more than 2 years.
13. Which hospital did you go to for this Laparotomy (Operation for stomach, appendix, intestinal disease)?
    - Same as above hospital mentioned in preference
    - Different Hospital
14. If it was a different hospital, what is the name of the hospital you went to?

    ______________________________
15. What is a famous landmark near this hospital

    ______________________________
16. What was the reason to choose this hospital?
    - Excellent doctors, nurses, and staff
    - Affordable treatment
    - Free medicines available
    - Focused on quality improvement
    - Referred by a friend/relative who also got treated here
    - My previous experience of treatment here was good
    - Referred by someone
    - Other reason
    - Don’t know
    - No answer
17. If other reason, please explain

    ______________________________
18. After reaching the hospital, how much time did you wait before you were seen by a doctor?

    ______________________________
19. Quality at hospital

| | Not satisfied at all | Satisfied | Very satisfied |
|---|---|---|---|

| | | | |
|---|---|---|---|
| Were you satisfied that the doctor listened to you attentively? | | | |
| Were you satisfied with the doctor's knowledge and experience? | | | |
| Were you satisfied that the doctor talked to you with respect? | | | |
| Were you satisfied with the amount of time doctor spent with you? | | | |
| Overall, how satisfied were you with your experience at this hospital? | | | |

20. What is the patient's condition now?
    - Completely treated
    - Short term complications
    - Long term complications
    - Death of the patient

**<u>Part 6: Specialized Surgery</u>** (surgeries such as cancer surgery, kidney, bladder surgery, or plastic surgery)

Record ID:

_____________________________

Data Entered by:

_____________________________

***Part 6a: Access to preferred hospital***

1. If anyone in the family would need a Specialized Surgery (surgeries such as cancer surgery, kidney, bladder surgery, or plastic surgery), which hospital would you prefer to go to? ______________________________
2. What is a famous landmark near the hospital. ______________________________
3. Out of the following 9 modes of transport, how many methods would you use? Ambulance, public transport (bus/rickshaw), Car, Motorcycle, Bicycle, Boat, Animal transport, walking, being carried by someone else)
    - One
    - Two
    - Three

If one, skip question 5 and 6

If two, skip question 6

4. What would your first mode of transport be in order to reach the hospital
    - Ambulance
    - Public transport
    - Rickshaw
    - Own car
    - Taxi/rented car
    - Neighbour's car
    - Motorcycle
    - Bicycle
    - Boat
    - Animal transport
    - Walking
    - Being carried by someone else

5. What would your second mode of transport be in order to reach the hospital
    - Ambulance
    - Public transport
    - Rickshaw
    - Own car
    - Taxi/rented car
    - Neighbour's car
    - Motorcycle
    - Bicycle
    - Boat
    - Animal transport
    - Walking
    - Being carried by someone else
6. What would your third mode of transport be in order to reach the hospital
    - Ambulance
    - Public transport
    - Rickshaw
    - Own car
    - Taxi/rented car
    - Neighbour's car
    - Motorcycle
    - Bicycle
    - Boat
    - Animal transport
    - Walking
    - Being carried by someone else
7. How long do you have to wait for arrangement of transport?
    ______________________________
8. After arrangement of transport, how long will it take you to reach the above mentioned hospital?

______________________________

9. How much will the transport cost?
    - 0-100 PKR
    - 100-200 PKR
    - 200-300 PKR
    - 300-400 PKR
    - 400-500 PKR
    - 500-1000 PKR
    - >1000 PKR
10. Is this much amount of money always readily available to you?
    - Yes
    - No

***Part 6b: Past experience***

11. In the last three years, has any household member required a Specialized Surgery (surgeries such as cancer surgery, kidney, bladder surgery, or plastic surgery)?
    - Yes
    - No

If no, then skip to next section.

12. How long ago did they have the Specialized Surgery (surgeries such as cancer surgery, kidney, bladder surgery, or plastic surgery)?
    - Less than 1 year
    - Less than 2 years but more than 1 year
    - Less than 3 years but more than 2 years.
13. Which hospital did you go to for this Specialized Surgery (surgeries such as cancer surgery, kidney, bladder surgery, or plastic surgery)?
    - Same as above hospital mentioned in preference
    - Different Hospital
14. If it was a different hospital, what is the name of the hospital you went to?

    ______________________________
15. What is a famous landmark near this hospital

    ______________________________
16. What was the reason to choose this hospital?
    - Excellent doctors, nurses, and staff
    - Affordable treatment
    - Free medicines available
    - Focused on quality improvement
    - Referred by a friend/relative who also got treated here
    - My previous experience of treatment here was good
    - Referred by someone
    - Other reason
    - Don’t know
    - No answer

17. If other reason, please explain
___________________________
18. After reaching the hospital, how much time did you wait before you were seen by a doctor?
___________________________
19. Quality at hospital

| | Not satisfied at all | Satisfied | Very satisfied |
|---|---|---|---|
| Were you satisfied that the doctor listened to you attentively? | | | |
| Were you satisfied with the doctor's knowledge and experience? | | | |
| Were you satisfied that the doctor talked to you with respect? | | | |
| Were you satisfied with the amount of time doctor spent with you? | | | |
| Overall, how satisfied were you with your experience at this hospital? | | | |

20. What is the patient's condition now?
    - Completely treated
    - Short term complications
    - Long term complications
    - Death of the patient

**Part 7: Deaths in household**

Record ID:

___________________________

Data Entered by:

___________________________

1. Has any family member died in the last 3 years
    a. Yes
    b. No

If no, skip to next section

2. In the last three years, how many family members died?

Details of deaths

| | | 1 | 2 | 3 | 4 | 5 | 6 |
|---|---|---|---|---|---|---|---|
| How long ago did the household member die? | | | | | | | |
| | Less than 1 year | | | | | | |
| | More than 1 year but less than 2 years | | | | | | |
| | More than 2 years but less than 3 years | | | | | | |
| What was the household member’s sex and were they adult or children? | | | | | | | |
| | Male - adult | | | | | | |
| | Female - adult | | | | | | |
| | Male - child | | | | | | |
| | Female- child | | | | | | |
| Did the household member die while in labour? | | | | | | | |
| | Yes | | | | | | |
| | No | | | | | | |
| | Not applicable | | | | | | |
| If it was a child, were they normal in appearance at birth. Was he able to breast feed, urinate, and stool at birth? | | | | | | | |
| | Yes | | | | | | |
| | No | | | | | | |
| | Not applicable | | | | | | |
| Did the deceased household member experience any of the following signs or symptoms within one week before his death? | | | | | | | |
| | Trauma/injury | | | | | | |
| | Injury other than trauma | | | | | | |
| | Illness or loss of blood while giving birth | | | | | | |
| | Swelling or lump | | | | | | |
| | Defect after birth | | | | | | |
| | Abdominal distension or pain | | | | | | |
| | None of the above | | | | | | |
| Were any of these signs or symptoms observed after a trauma or accident? What kind of trauma or accident was it? | | | | | | | |
| | Car, bus, truck accident | | | | | | |
| | Motorcycle/ bike accident | | | | | | |
| | Accident while walking or cycling | | | | | | |
| | Gunshot | | | | | | |
| | Dagger/ crushing under heavy weight | | | | | | |
| | Animal bite | | | | | | |
| | Falling from height | | | | | | |
| | Fire/explosion/Bomb blast | | | | | | |
| | Burn | | | | | | |
| Did the deceased go to any spiritual or traditional healer? | | | | | | | |
| | Yes | | | | | | |
| | No | | | | | | |
| Did the deceased go to any clinic or hospital to consult regarding his illness before his death? | | | | | | | |
| | Yes | | | | | | |

| | | 1 | 2 | 3 | 4 | 5 | 6 |
|---|---|---|---|---|---|---|---|
| | No | | | | | | |
| What kind of treatment did the deceased household member undergo? | | | | | | | |
| | No treatment | | | | | | |
| | Major surgery | | | | | | |
| | Minor surgery | | | | | | |
| What was the reason for not visiting clinic or hospital and consulting a doctor or nurse? | | | | | | | |
| | No money for treatment | | | | | | |
| | No money or facility for transport to hospital | | | | | | |
| | No time left to arrange | | | | | | |
| | No trust on treatment or fear of treatment | | | | | | |
| | No doctor or hospital facility available | | | | | | |
| | No need for surgery | | | | | | |
| Where did the deceased household member die? | | | | | | | |
| | Home | | | | | | |
| | Hospital/clinic | | | | | | |
| | Elsewhere | | | | | | |

What was the reason for going to Traditional/Spiritual healer?

1. ______________________________________________________________________________
   ___________________________________________________________
2. ______________________________________________________________________________
   ___________________________________________________________
3. ______________________________________________________________________________
   ___________________________________________________________
4. ______________________________________________________________________________
   ___________________________________________________________
5. ______________________________________________________________________________
   ___________________________________________________________
6. ______________________________________________________________________________
   ___________________________________________________________

Summarize story of each deceased household members death in your own words.

1. ______________________________________________________________________________
   ___________________________________________________________
2. ______________________________________________________________________________
   ___________________________________________________________
3. ______________________________________________________________________________
   ___________________________________________________________
4. ______________________________________________________________________________
   ___________________________________________________________
5. ______________________________________________________________________________
   ___________________________________________________________

6. ______________________________________________________________________________
______________________________________________________________

**Part 8: Financial status assessment**

1. What occupation is held by the person who makes the most money in your household (primary breadwinner?)?
   - Unemployed
   - Housewife
   - Home maid
   - Farmer
   - Owned business
   - Govt service employee
   - Private service employee
   - Laborer
   - Other
2. If other, please state
   _____________________________
3. How much does your household earn in an average month?
   _____________________________
4. How much money does your household spend on food to eat, water, juice or soda to drink?
   _____________________________
5. How much money does your household spend on livestock (including cows, pigs, goats, sheep, chickens etc.) every month?
   _____________________________
6. How much money does your household usually spend on health care, including medicines, fees for doctors or hospital visits, fees for traditional healers?
   _____________________________
7. How much does your household pay for your house/apartment in rent or mortgage or housing fees (if applicable)?
   _____________________________
8. How much money does your household spend on other household matters such as clothes, improvements to your house etc?
   _____________________________
9. How much money does your household spend on education per term for all the children you support, including school fees and books?
   _____________________________
