# Supplementary material for: Estimating access to surgical care: A community centered national household survey from Pakistan
Source: PLOS Glob Public Health. 2023 Nov 15;3(11):e0002130. doi: 10.1371/journal.pgph.0002130 (PMC10651040; doi:10.1371/journal.pgph.0002130)
Supplement: S1 Table — (DOCX) [file pgph.0002130.s002.docx]

**Supplemental Table 1**

| Supplemental Table 1: Design Effects (DEFF) for the accessibility estimates | | | | | | | |
| --- | --- | --- | --- | --- | --- | --- | --- |
|  |  | **All Procedures** | **Bellwethers** | **C-section** | **Laparotomy** | **OFR** | **Specialized Surgery** |
| *Overall* |  | 4.79 | 4.28 | 2.91 | 4.02 | 3.7 | 4.77 |
| *Province* |  |  |  |  |  |  |  |
|  | Punjab | 6.03 | 5.86 | 4.79 | 5.42 | 5.28 | 6.13 |
|  | Sindh | 3.33 | 2.53 | 1.86 | 1.78 | 2.08 | 3.08 |
|  | KPK | 1.29 | 1.55 | 1.85 | 1.91 | 1.69 | 1.28 |
|  | Balochistan | 0.52 | 0.47 | 0.55 | 0.5 | 0.53 | 0.52 |
|  | Islamabad CT | 0.000 | 0.000 | 0.000 | 0.000 | 0.000 | 0.000 |
| *Urban vs Rural* |  |  |  |  |  |  |  |
|  | Rural | 2.64 | 3.09 | 2.5 | 2.86 | 2.98 | 3.38 |
|  | Urban | 3.37 | 2.32 | 1.46 | 1.98 | 2.21 | 2.47 |
| *Household consumption quintile* |  |  |  |  |  |  |  |
|  | 1st quintile | 2.670 | 2.390 | 1.820 | 2.420 | 2.230 | 2.650 |
|  | 2nd quintile | 2.020 | 2.500 | 2.320 | 2.330 | 2.020 | 2.000 |
|  | 3rd quintile | 1.760 | 1.720 | 1.100 | 1.770 | 1.260 | 1.660 |
|  | 4th quintile | 2.750 | 2.380 | 0.950 | 2.070 | 1.540 | 2.810 |
|  | 5th quintile | 1.890 | 1.410 | 1.440 | 1.390 | 1.370 | 2.040 |
