# Supplementary material for: Estimating access to surgical care: A community centered national household survey from Pakistan
Source: PLOS Glob Public Health. 2023 Nov 15;3(11):e0002130. doi: 10.1371/journal.pgph.0002130 (PMC10651040; doi:10.1371/journal.pgph.0002130)
Supplement: Attachment — Submitted filename: Response to Reviewers.docx [file pgph.0002130.s003.docx]

# Response to Reviewers

Response to General changes requested

1. **Competing interests to be stated clearly. If you have no competing interests to declare, please state "The authors have declared that no competing interests exist".**

Addressed

2. **Amend detailed Financial Disclosure statement
   a. State the initials, alongside each funding source, of each author to receive each grant.**

Addressed
**b. State what role the funders took in the study. If the funders had no role in your study, please state: “The funders had no role in study design, data collection and analysis, decision to publish, or preparation of the manuscript.”**

Addressed
**c. If any authors received a salary from any of your funders, please state which authors and which funders. If you did not receive any funding for this study, please simply state: “The authors received no specific funding for this work.”**

Not applicable

3. **Please provide separate figure files in .tif or .eps format only and remove any figures embedded in your manuscript file. Please also ensure that all files are under our size limit of 10MB.**

Addressed

4. **Fig 1: please (a) provide a direct link to the base layer of the map (i.e., the country or region border shape) and ensure this is also included in the figure legend; and (b) provide a link to the terms of use / license information for the base layer image or shapefile. the terms of use for your map base layer must be compatible with our CC-BY 4.0 license.**

<https://data.humdata.org/dataset/cod-ab-pak> ;The link has the main data page and a metadata page; the license mentioned is Creative Commons for Intergovernmental Organisations. Details about the sharability and use of data for the license are given on this link: <https://data.humdata.org/faqs/licenses> . It states “Under the CC BY-IGO license, you are free to share (copy and redistribute the material in any medium or format) and or adapt (remix, transform, and build upon the material) for any purpose, even commercially.“

Response to Editor’s comments

1. **How well do lay people in the community know whether they have reliable access to a particular type of surgical procedure?**

Procedures were described to the respondents in their local language using lay people terms. Rather than determining 2-hour access using a dichotomous question, the respondents were asked about the hospital they would go to for the particular procedure, what transport they would use to travel, and based on the transport used, how long it would take to reach the preferred facility. The study team reviewed the responses on a regular basis for plausibility. If clarifications were needed, the data collectors were contacted for clarification.

2. **Could you please include the full interview tool as an appendix please?**

Attached

3. **The numbers in table 2 could be reduced to 2 decimal places to improve readability , or otherwise would me more easily read if converted to percentages with one decimal place.**

Converted to percentages

4. **Please don't use abbreviations like "C-section", however obvious the abbreviation may be to you, without first spelling the abbreviation out in full. Another example is "LMIC" which could be taken to mean lower-middle income country or low- and middle-income country. There are other examples in the text (DALY).**

We have detailed the abbreviations. Thank you for pointing out the issue.

5. **In the abstract you write "Pakistan is an LMIC in South Asia". It is not specified whether you mean lower-middle income country or low- and middle- income country.**

Addressed

6. **"Sub-Saharan" should be "sub-Saharan"**

Addressed

7. **Perhaps "The Lancet Commission on Global Surgery (LCoGS) estimates 5 billion people lack access to timely, safe, quality, and affordable surgical care" should be written in the past tense**

Addressed

8. **"While population access to C-sections is found to be above 80%, population access to Laparotomy, OFR, and specialized surgery is suboptimal." I note that 80% access to Cesarean section is still not "optimal" even if it meets your agreed target.**

Agree with the comment. Change made to wording

9. **The discussion section is rather long and could be made more concise.**

We have reviewed the discussion and made it more concise.

10. **Your link for the data does not seem to work - can you check this please?**

This link should work, if not, please let me know: https://dataverse.harvard.edu/privateurl.xhtml?token=bc6cbda9-c33c-4394-9916-6f893f3ba593

Response to reviewer 1 (Wesley Rajaleelan) comments:

1. **"Poor surgical care service availability at secondary-level hospitals that are supposed to be the front line for emergency surgical services is one of the main reasons for the rural disparity, Despite being designated as centers for essential surgery, surveys of rural secondary-level hospitals have shown that up to 71% do not have an anesthetist", I am curious to know if there are any bridging courses available for medical officers to administer spinal anesthesia for acute emergencies like a c section?**
2. **To mitigate the constraint of specialist providers, the de facto standard of care in many nations is task-sharing of anesthesia provision, which is estimated to occur in as many as 119 countries, across all World Bank income groups. In India, medical officers, who are non-specialist medical graduate physicians, have a precedent of delivering anesthesia care in rural hospitals. Medical officers are incentivized to work in non-urban healthcare settings in a number of ways inclusive of subsidized medical education at government medical colleges or as a mandatory requirement to fulfil prior to postgraduate specialization. Just curious to know if such a program is available in Pakistan, as many of the described staff shortages can be phased out?**

Thank you for bringing up this important limitation in the delivery of essential surgical services. The reviewer is correct in pointing out that there is a paucity of anesthetist at the rural hospital level in most LMIICs. Pakistan is a little different, on paper at least, due to two facts. The first is that there are multiple levels and tracks of anesthesia providers in the country, between the federal, provincial, and even private credentialing pathways. These can vary from a full "residency" of 4 years (FCPS), modified practical residencies of 2 years (MCPS), and even certification/diploma programs of 1 year (DA). Some of these are even open to non-physicians like technicians. In fact, in many settings, anesthesia technicians (college graduates who have done a 2-year diploma course) perform general anesthesia with an endotracheal tube and spinal anesthesia "unofficially." The second point differentiating Pakistan is that the vast majority of rural hospitals are in the public sector, and officially have an anesthesia provider assigned to locations that have surgeons (OB or otherwise) assigned. However, it must be recognized that this is frequently not the case on the ground, where anesthesia providers often cover multiple locations in the private sector as well, or only come on limited days to their assigned rural hospitals if at all. Furthermore, the level of anesthesia providers can be very variable due to the first point. The norm in most smaller hospitals is that surgeons (OB or otherwise) have standing relationships with an anesthesia provider from the area (usually at the diploma/certificate level who might or might not be a physician), and bring them along for every case that they do. In a limited number of circumstances, surgeons still perform spinal anesthesia themselves which they have learnt from on-the-job training.

Response to Reviewer 2:

1. **Methodology: Would an ordinary member of the community be expected to know whether services for laparotomy and fractures are available – or was this information matched between information from the tehsils. This could be better detailed in the methodology. This will determine whether the data is fully accurate or based on the perspectives of the people interviewed, rather than availability of services.**

Procedures were described to the respondents in their local language using lay people terms. Rather than determining 2-hour access using a dichotomous question, the respondents were asked about the hospital they would go to for the particular procedure, what transport they would use to travel, and based on the transport used, how long it would take to reach the preferred facility. The study team reviewed the responses on a regular basis for plausibility. If clarifications were needed, the data collectors were contacted for clarification.

2. **Results – reported access was lower for laparotomy, then caesarean section – was there any way of ensuring that the patients clearly understood the definitions?**

Caesarean-section per se is a commonly used term even in rural and low income populations in Pakistan. Moreover, the clear differentiating factor remains the birth of a baby as a clear outcome for this procedure, and hence we have never faced any challenges in communicating the difference between C-section vs Laparotomy in our 30 year experience of working in Pakistan in both clinical and public health settings.

3. **Table 4 – this is very interesting. Again is this reported? Is it accurate. Could it be assumptions. Was there an attempt to verify. In many LMIC’s with lower levels of education, communities may not always know what services are available even in their local hospitals, especially when it comes to specialised surgical services.**

This is a very valuable perspective. During the interview, our question was phrased in a way so as to elicit the household’s preferred facility for the 4 different categories of surgeries. This was in effect a way of gauging patterns of “perceived access” and aimed to document the bottom line of where the household will go for the given surgical procedure. It is not possible to verify the depth of information that a household may have about the available options, but this question did not aim to study the availability of facilities, instead focused on facilities preferred by the household. So in essence, this question was aimed at understanding from the perspective of the population about where they choose to go, rather than a factual documentation of availability of services in hospitals in that area. We have tried to address this in the limitation section of the manuscript.

This study aimed to estimate access through the perceptions and preferences at the household level and did not assess service availability in nearby hospitals. Therefore, there may be options available within the 2-hour access perimeter that may not be known to the household members.

4. **Discussion – line 1, I would recommend that you add – ‘according to a household survey’.**

Edited as suggested.

5. **Discussion paragraph 2: Access to essential surgery does not equal access to specialist surgeons. It is very common is LMICs that surgeries are not done by specialists, even complex and complicated surgeries. Even in tertiary hospitals the primary surgeons is usually a registrar or resident who calls for help when in need of a specialist. This is not surprising in the context of an LMIC. This must be explained in the context of this country and not generalised so that we know who is actually doing the operations.**

The questions asked in this survey were aimed at the procedure and not the surgeon. Hence, at no point have we attempted to assess the specialization level or even credentials of the surgeons. The reason for this is that the qualification of the surgeon is not something that most patients are able to evaluate or understand. Hence, we have only asked about the procedures.

6. **It does appear that the solution to access may not be increasing volume – if the country needs to go from 89/100 000 to 5000/100 000. The feasibility of the recommendation by the Lancet Commission on Global Surgery needs to stated with caution. Reducing the need for surgery is a strategy that may go hand in hand with increasing the surgical volume and surgeons.**

This is a useful perspective and duly noted. However, the scope of our study does not extend to prevention of surgical conditions and hence we do not think it appropriate to address this aspect in our discussion, especially given word count constraints.

7. **Discussion final paragraph: Even with the proposed institutionalisation of indicators the interpretation of data needs to be taken in context of the local setting. The concern with pushing for a focus on numbers is that generalisations that lead to misinterpretation of data can be made – e.g. what does 74.8% compared to benchmark of 80% really say to us about access to surgical care, especially if we do not know who is operating (specialist vs non-specialist) and what the outcomes of the patients are (dead/alive and healthy/severe complications). Please expand more on what we really learn from this finding – and why the idea of a national indicator would improve access.**

This is an important aspect, we have added a sentence to focus on the importance of quality and safety of surgical procedures particularly as we attempt to scale up services. However, given the editorial request to shorten the discussion section, we are unable to expand on this more.
