# Supplementary material for: Estimating access to surgical care: A community centered national household survey from Pakistan
Source: PLOS Glob Public Health. 2023 Nov 15;3(11):e0002130. doi: 10.1371/journal.pgph.0002130 (PMC10651040; doi:10.1371/journal.pgph.0002130)
Supplement: Attachment — Submitted filename: Responses 7-2-23.docx [file pgph.0002130.s004.docx]

1. Please amend your online detailed Financial Disclosure statement. This is published with the article. It must therefore be completed in full sentences and contain the exact wording you wish to be published.

   a) State the initials, alongside each funding source, of each author to receive each grant. For example: "This work was supported by the National Institutes of Health (####### to AM; ###### to CJ) and the National Science Foundation (###### to AM)."
   b) State what role the funders took in the study. If the funders had no role in your study, please state: “The funders had no role in study design, data collection and analysis, decision to publish, or preparation of the manuscript.”

   Thank you for the feedback. This has been addressed.
2. Please ensure that the funders and grant numbers match between the Financial Disclosure field and the Funding Information tab in your submission form. Note that the funders must be provided in the same order in both places as well.

   Thank you for the feedback. This has been addressed.
3. Please update your online Competing Interests statement. If you have no competing interests to declare, please state: “The authors have declared that no competing interests exist.”

Thank you for the feedback. This has been addressed.

4. We note that your Data Availability Statement reads: "The datasets generated and/or analyzed during the current study are available at [https://dataverse.harvard.edu/privateurl.xhtml?token=bc6cbda9-c33c-4394-9916-6f893f3ba593"](https://dataverse.harvard.edu/privateurl.xhtml?token=bc6cbda9-c33c-4394-9916-6f893f3ba593%22)

   Unfortunately, this information yields no publicly available results. Please confirm that you have provided the correct DOI/link and that your data is set to be publicly available. We will hold your manuscript in the meantime. We hope to hear from you soon with updated information.

Thank you for the feedback. This has been addressed. Please try this link. <https://dataverse.harvard.edu/dataset.xhtml?persistentId=doi%3A10.7910%2FDVN%2F1AQCD4&version=DRAFT>

5. Please ensure that you refer to Figure 1 in your text as, if accepted, production will need this reference to link the reader to the figure. Please ensure that you refer to Table 1 in your text as, if accepted, production will need this reference to link the reader to the table.

Thank you for the feedback. Both of the above concerns have been addressed.
