# Supplementary material for: Estimating access to surgical care: A community centered national household survey from Pakistan
Source: PLOS Glob Public Health. 2023 Nov 15;3(11):e0002130. doi: 10.1371/journal.pgph.0002130 (PMC10651040; doi:10.1371/journal.pgph.0002130)
Supplement: Attachment — Submitted filename: Rebuttal.docx [file pgph.0002130.s005.docx]

**Rebuttal**

**Journal requirements**

Please review your reference list to ensure that it is complete and correct. If you have cited papers that have been retracted, please include the rationale for doing so in the manuscript text, or remove these references and replace them with relevant current references. Any changes to the reference list should be mentioned in the rebuttal letter that accompanies your revised manuscript. If you need to cite a retracted article, indicate the article’s retracted status in the References list and also include a citation and full reference for the retraction notice.

Response: No changes made to reference list.

**Reviewer 3 comments**

*Introduction*
1. First paragraph: the reference cited reported a range of 28-32%. And the author referring to results of another author. It may be corrected to “Shrime reports are health care providers' estimates, which they reported to be 28-32%”.

Response: Thank you for the feedback. The wording has been revised.

2. To suffice English language in publications you may need to change/rephrase the sentence beginning with the majority of these…… “Injuries (77%), maternal and neonatal conditions (14%), and gastrointestinal conditions contributed to the majority of preventable mortality”.

Response: Thank you for the feedback. The wording has been revised.

*Methodology*
1. Specify the duration of interruption from a Covid-19 pandemic.

Response: Thank you for the feedback. Duration has been specified.

2. Data collection- Identify members of the household who was/were interviewed. Some questions on household consumption may be sensitive in some cultures or require a leading member of the household.

Response: Thank you for the feedback. We have revised the manuscript and mentioned that the heads of households were interviewed.

*Results*
1. After Table 1, the second table needs rephrasing as the numbers are too close. It may read C-sections procedures had the highest access of 88.7%, followed by OFR at 81% and laparotomy at 79%"
In table two, the column with All procedures, does it include specialized and Bellwether procedures? If so, it needs to be specified in such rather than All procedures.

Response: Thank you for the feedback. The wording has been revised.

2. Opening statements before Table 3- To my understanding this was a household survey, avoid reporting population access. And provide a summary of the table. Rephrase "Highest access to Bellwether procedures is observed in Islamabad CT (92.9%), and Punjab (81%). In contrast, Balochistan had the lowest rate (28.6%) (Table 2).

Response: Thank you for the feedback. The wording has been revised.

3. You may choose one method of statistical significance, either a CI or P value.

Response: Thank you for the feedback. However, we are opting for both CI and p-values for the readers’ convenience.


*Discussion*
Second last paragraph- This sounds like a study limitation

Response: Thank you for the feedback. The wording has been revised.

Good work to authors. Some adjustments will polish this work even better.

Response: We appreciate your time and effort in helping us improve our manuscript.
