# Supplementary material for: Estimating access to surgical care: A community centered national household survey from Pakistan
Source: PLOS Glob Public Health. 2023 Nov 15;3(11):e0002130. doi: 10.1371/journal.pgph.0002130 (PMC10651040; doi:10.1371/journal.pgph.0002130)
Supplement: Attachment — Submitted filename: Rebuttal.docx [file pgph.0002130.s006.docx]

Editor Comments:

Thanks for addressing the issues raised by the reviewers. Please address the following issues:

1. Please explain how informed consent was obtained from the participants and the institutions where ethical approval were obtained.

***Response:*** *Ethical approval to conduct the study was received from the Interactive Research and Development Institutional Review Board (IRD IRB 2018 05 006) and the Harvard Medical School Institutional Review Board. The Ministry of National Health Service Regulation and Coordination, Government of Pakistan, and the provincial departments of health issued letters of support to conduct the study.*

*Informed consent was collected on paper by health worker from the head of the household after explaining the risks and benefits of participation in the study in their native language.*

*This information has been added to the manuscript in the methodology section.*

2. You have reported that "We assumed an intra-cluster correlation coefficient (ICC) of 1/6". What is 1/6? ICC can have a value between 0-1 and commonly is between 0.01 and 0.02 . Please correct this.

***Response:*** *ICC can vary from 0-1 and is a measure of homogeneity within a cluster.*

*We assumed access to surgical care within a cluster is spatially correlated, therefore a higher than normal intra-cluster cluster coefficient (ICC) of 0.167 was used similar to ICC recommended for immunization coverage surveys by WHO*

*This information has been added to the manuscript in the methodology section with reference to the “WHO vaccination coverage cluster surveys: reference manual” .*

*Reference:* [***WHO Vaccination coverage cluster surveys: reference manual. Accessed September 29, 2023. https://healthcluster.who.int/publications/m/item/who-vaccination-coverage-cluster-surveys-reference-manual***](https://d.docs.live.net/53a536522b15649e/Desktop/Household%20survey%20manuscript/Revision%209-29-23/WHO%20Vaccination%20coverage%20cluster%20surveys:%20reference%20manual.%20Accessed%20September%2029,%202023.%20https:/healthcluster.who.int/publications/m/item/who-vaccination-coverage-cluster-surveys-reference-manual)

Journal Requirements:

1. Please review your reference list to ensure that it is complete and correct. If you have cited papers that have been retracted, please include the rationale for doing so in the manuscript text, or remove these references and replace them with relevant current references. Any changes to the reference list should be mentioned in the rebuttal letter that accompanies your revised manuscript. If you need to cite a retracted article, indicate the article’s retracted status in the References list and also include a citation and full reference for the retraction notice.

***Response:*** *No retracted article has been referenced. We added one reference to the list which is listed below:*

*11.* [***WHO Vaccination coverage cluster surveys: reference manual. Accessed September 29, 2023. https://healthcluster.who.int/publications/m/item/who-vaccination-coverage-cluster-surveys-reference-manual***](WHO%20Vaccination%20coverage%20cluster%20surveys:%20reference%20manual.%20Accessed%20September%2029,%202023.%20https://healthcluster.who.int/publications/m/item/who-vaccination-coverage-cluster-surveys-reference-manual)

2. While revising your submission, please upload your figure files to the Preflight Analysis and Conversion Engine (PACE) digital diagnostic tool, https://pacev2.apexcovantage.com/. PACE helps ensure that figures meet PLOS requirements. To use PACE, you must first register as a user. Registration is free. Then, login and navigate to the UPLOAD tab, where you will find detailed instructions on how to use the tool. If you encounter any issues or have any questions when using PACE, please email PLOS at figures@plos.org. Please note that Supporting Information files do not need this step.

***Response:*** *Figure uploaded to pace and revised figure uploaded as requested.*
